# Optimization of CASA-Mot Analysis of Donkey Sperm: Optimum Frame Rate and Values of Kinematic Variables for Different Counting Chamber and Fields

**DOI:** 10.3390/ani10111993

**Published:** 2020-10-29

**Authors:** Sabrina Gacem, Jaime Catalán, Anthony Valverde, Carles Soler, Jordi Miró

**Affiliations:** 1 Equine Reproduction Service, Department of Animal Medicine and Surgery, Faculty of Veterinary Sciences, Autonomous University of Barcelona, 08193 Bellaterra, Spain; swp.sabrina.gacem@gmail.com (S.G.); dr.jcatalan@gmail.com (J.C.); 2 Costa Rica Institute of Technology, School of Agronomy, San Carlos Campus, 223-21001 Alajuela, Costa Rica; anvalverde@itcr.ac.cr; 3 Departamento de Biología Celular, Biología Funcional y Antropología Física, Universitat de València, 46100 Burjassot, Valencia, Spain

**Keywords:** frame rate, drop displacement chambers, capillary loaded chambers, chamber depth, field, sperm dilution

## Abstract

**Simple Summary:**

A reliable sperm motility exam is important for semen analysis and breeding soundness examination. Different parameters can affect the Computer Assisted Sperm Analysis (CASA) motility results. Today, new high-resolution cameras and different chambers are introduced to CASA systems, and protocol optimization is required to render the estimation results for donkey sperm. The objective of this study is the optimization of the conditions used for donkey semen motility analysis with CASA-Mot by defining the optimum frame rate for different chamber types. Additionally, to study the effect of different chamber types, chamber field and sperm dilution on the sperm kinematic parameters with higher frame rates are examined.

**Abstract:**

In order to optimize the donkey sperm motility analysis by the CASA (Computer Assisted Sperm Analysis)-Mot system, twelve ejaculates were collected from six jackasses. Capillary loaded chamber (CLC), ISAS^®^D4C depths 10 and 20 µm, ISAS^®^D4C Leja 20 and drop displacement chamber (DDC), Spermtrack^®^ (Spk) depths 10 and 20 µm were used. Sperm kinematic variables were evaluated using each chamber and a high-resolution camera capable of capturing a maximum of 500 frames/second (fps). The optimum frame rate (OFR) (defined according to curvilinear velocity—VCL) was dependent on chamber type. The highest OFR obtained was 278.46 fps by Spk20. Values for VCL, straight-line velocity (VSL), straightness (STR), amplitude of lateral head displacement (ALH) and beat cross frequency (BCF) were high in DDC and 10 µm depth. In both DDC 10 and 20 µm, the sperm velocities (VCL, VSL, VAP) and ALH values decreased significantly from the centre to the edges, while Wobble and BCF increased. No defined behavior was observed along the CLC. However, all the kinematic variables had a higher value in a highly concentrated sample, in both chamber types. In conclusion, analyzing a minimum of nine fields at 250 fps from the centre to the edges in Spk10 chamber using a dilution of 30 × 10^6^ sperm/mL offers the best choice for donkey computerised sperm motility analysis.

## 1. Introduction

The domestic donkey (*Equus asinus*) is one of the two domestic species of the genus Equus along with the horse (*Equus caballus*) [1]. In developed zones, donkeys have suffered a significant decrease due to industrialization and mechanization of agriculture. However, in recent years, there has been an increase in donkey product interest: milk, meat or skin [2]. Donkey farming is expanding and the research interest about donkey production and reproduction optimization is increasing [3,4]. This implies a more complete knowledge of their semen quality and general reproductive characteristics especially for achieving a productive-assisted reproduction [5].

Semen quality can be defined upon certain criteria performing certain tests, such as motility, concentration and morphology [5,6,7]. Nonetheless, from all those tests, sperm motility is commonly considered the most significant parameter for breeding soundness examination [8,9]. Usually, motility is analysed subjectively looking for just the total and progressive motility, but in the last few decades, an objective method—CASA (Computer Assisted Sperm Analysis)—was introduced and is now available and used widely by veterinarians and laboratories [10]. CASA-Mot technology is based on the computational reconstitution of sperm trajectory from image sequences; the last ones are captured by a video camera mounted on a microscope. Then, the sperm sequences are automatically analysed by the computer in a concise time [11]. Moreover, CASA-Mot systems provide a battery of kinematic quantitative parameters that define the sperm cell motility rather than the progressivity [12].

A critical review of the literature revealed that no standard practices have been embraced or recommended by professional societies in the case of donkey samples and thus, no defined protocols are followed within or across CASA-Mot instruments [13]. In fact, in other species, it has been proved that the accuracy and the sensitivity of the measurements obtained with CASA-Mot systems can be affected by different factors, such as the mathematical algorithms, suspending medium, sample concentration, frame rate, chamber type and depth, hardware, and instrument settings [14]. Usually, for the donkey semen assessment within CASA, we use the same setting and protocol as horses. However, comparative studies in both species have identified differences in reproductive strategy as well as in the sperm form and function [15,16]. Effectively, the donkey testis is bigger and has been proved to have more efficient spermatogenesis than a stallion [17,18,19,20]. In effect, donkey sperm heads are smaller with a larger mid-piece than the stallion resulting in differences in motility patterns [21]. It was reported that donkey spermatozoon is faster than the horse when using the same CASA set up [22]. However, we need to consider those differences in the definition of the most adequate setting when analysing donkey sperm.

On the other hand, in recent years, the development of high-resolution cameras, the improvement of informatic systems and the development of specific chambers to analyze sperm motility have changed the published data completely on sperm motility patterns in different species by CASA-Mot systems.

The objective of this study was to standardise the method of sperm motility assessment in the donkey, by first defining the optimal frame rate (OFR) based on VCL (curvilinear velocity) data for different counting chambers for the use in CASA-Mot system. Secondly, we analyse the kinematic variables at OFR in different chamber types and depths (including the considered counting area). Finally, an investigation of the effect of semen dilution on donkey sperm motility parameters is carried out.

## 2. Materials and Methods

### 2.1. Animals Used and Ethics Statement

The experiment was carried out on six Catalan donkeys with two ejaculates for each one. All males were aged between 3 and 20 years old and they are known for their successful fertility. Animals involved in the study were housed at the Equine Reproduction Service, Autonomous University of Barcelona (Bellaterra, Cerdanyola del Valles, Spain) at a Europe-approved equine semen collection centre (authorization number: ES09RS01E). The centre operates under strict protocols of animal welfare and health control. All jackasses were semen donors, which were housed in an individual paddock at the centre. Semen has been collected under CEE health conditions (free of Equine Arteritis, Infectious Anaemia and Contagious Metritis). It is important to note that the service runs under the Catalonia Regional Government’s approval (located in Spain) and no manipulations to the animals other than semen collection were carried out. The Ethics committee of this institution indicated that no further ethical approval was required. Additionally, all the animals received a standard diet (with mixed hay and basic concentrate) and were provided with water ad libitum. Three times a week, donkeys underwent regular semen collection once a day under the same conditions and samples were collected throughout the year.

### 2.2. Semen Preparation

The semen was collected manually using an artificial Hannover vagina (Minitüb GmbH, Tiefenbach, Germany) with an in-liner nylon filter to eliminate the gel fraction. Once semen collected, the volume was recorded, then was immediately diluted 1:5 (*v*/*v*) in a skim-milk-based semen extender (Kenney) [23] and allowed in 50 mL conical tubes. Morphological abnormalities and viability were determined by bright field microscopy (mag. ×1000) when examining 200 cells after smear staining with Eosin–Nigrosin. Total sperm concentrations were determined using a Neubauer Chamber (Paul Marienfeld GmbH and Co. KG, Lauda-Königshofen, Germany); counting was performed in triplicate using a phase-contrast microscope (mag. ×20).

### 2.3. Semen Dilution

The sperm concentration of semen diluted previously was reevaluated and adjusted to obtain two groups: the first one with a high concentration (80 × 10^6^ spz/mL) and the second, low concentrated, (30 × 10^6^ spz/mL) in order to analyze the effect of concentration on sperm motility.

### 2.4. Counting Chambers and Loading Technique

**Figure 1:**
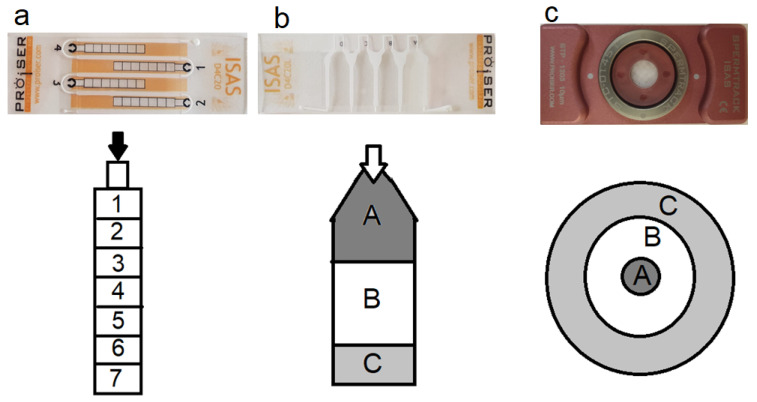
The different chambers types and forms used and the filed analyzed. (**a**): Disposable chamber D4C10, D4C20. (**b**): Disposable chamber D4C20L and (**c**): Reusable chambers SpK10, SpK 20. The arrow demonstrates the place of drop deposition. Numbers (1–7) and characters (A, B, C) inside the chambers demonstrate different capture fields.

Five commercial counting chambers (all from Proiser R + D S.L., Paterna, Spain) were used: (1) three disposable, capillary loaded chambers ISAS^®^D4C having a fixed cover-slide attached by glue, ISAS^®^D4C10, ISAS^®^D4C20 (hereafter D4C10 and 20) of 10 and 20 μm depth (Figure 1a) and ISAS^®^D4C20L (hereafter D4CL20) of 20 µm depth (Figure 1b). (2) two reusable drops displacement chambers Spermtrack^®^ (Proiser R + D, Paterna, Spain) having a separate cover slide, Spermtrack^®^10 and Spermtrack^®^20 (hereafter Spk10 and 20) of 10 and 20 µm depth (Figure 1c).

The samples were well homogenized just before being charged in the chambers. All chambers were loaded with the adequate technique and quantity (Figure 1) of semen as recommended by the manufacturer. When using the Spermtrack^®^ chambers, the covers were rapidly but gently put in place to achieve a homogenous distribution of the sample. The other chambers were loaded by depositing the sperm sample in the loading area. Then, the sperm travels by capillarity into the different areas of the chamber. Chambers were maintained on a thermo-plate for 15 s at 37 °C to prevent heat shock and allowing the fluid to cease permitting correct observation. For the analysis, the order of chambers used was randomized to avoid the effect of incubation time.

To study the effect of the field location on sperm motility, 7 fields were identified longitudinally in D4C10 and 20 (Figure 1c). However, in D4C20L two captures were made in each zone A, B and C from the proximal position to the distal position as presented in Figure 1b. Finally, in Spermtrack^®^ chamber different fields were captured from the center to the edges as shown in Figure 1c. An average of 500 sperm was captured per field.

### 2.5. CASA-Mot Analysis

Objective motility assessment was performed using ISAS^®^ v1.2 CASA-Mot system (Proiser R + D, Paterna, Spain) combined with a UOP200i microscope (Proiser R + D) equipped with a negative phase contrast 10× objective (AN 0.25) and an MQ003MG- CM digital camera (Proiser R + D S.L.) which was capable of capturing a maximum frame rate of 500 per second. The final resolution was 0.48 µm/pixel in both x- and y-axis. The system was set with particle area between 4 and 70 µm^2^ and connectivity set value of 6 µm. All samples analysis was performed by the same technician to avoid errors related and biases.

Sequences were captured at 500 fps and recorded during 3 s in different fields. For the study, these original videos were later segmented into 25, 50, 100, 150, 200 and 250 fps working videos, using the following command: [echo off: set fps = 25, 50, 75, 100, 150: for %%i in (.Ä*.avi) do (set fname = %%~ni) & call: encodeVideo; goto eof: encodeVideo: ffmpeg.exe -i%fname%.avi -r %fps% -clibx264 -preset slow -qp 0”%fname%_(%fps%fps).avi”; goto eof].

The following kinematic parameters were considered for this study: the sum of the distances between each measured sperm position divided by the analysis time (VCL, µm/s), the straight-line distance between the first and last sperm position divided by the analysis time (VSL, µm/s), the average path velocity is the time-averaged velocity of a sperm head along its average path (VAP, µm/s), the wobble is a measure of oscillation of the actual path about the average path (WOB = VAP/VCL, dimensionless), the straightness is a measure of the linearity of the average path (STR = VSL/VAP, dimensionless), the linearity of forward progression, the linearity of the curvilinear path (LIN = VSL/VCL, dimensionless), the average distance of the sperm head from the average sperm-swimming path where the average path (ALH, µm) and the beat cross frequency the number of lateral oscillatory movements of the sperm head around the mean trajectory (BCF, Hz).

### 2.6. Calculating the Optimum Frame Rate

Optimal frame rates were determined from the VCL of the sperms using point-to-point reconstructions of their trajectories at each tested FR. For this, the results were subjected to exponential regression analysis:

y = β.α exp (−β/x)(1)

where y is the VCL, x is the FR, α is the asymptotic level, β is the rate of increase to the asymptote and exp based on the natural logarithm. The biological meaning of the formulae is that the asymptotic level (α) represents the maximum achievable when the FR is above the threshold value. The threshold level is conventionally calculated as the FR needed to obtain 95% of the maximum value. The rate of the approach to the asymptote represents the dependence on the curve on the FR; a higher value of β indicates high growth of the VCL as FR increases and vice versa. There is no substantial increase in the VCL with the increase of FR which represents α the asymptotic value (at least 95% of the maximum VCL has been achieved). The rate of approach to the asymptote represents the dependence of the curve on the FR, thus, a high β value indicates an increase in VCL with increasing FR and vice versa.

Although, the maximum image captured by the camera was 500 f/s. There is no software that can analyse the huge amount of data generated by capture frequency higher than 250 f/s. Therefore, given the OFR for all the studied chambers was either virtually identical to 250 f/s or >250 f/s. The following experiments were performed at 250 f/s for every chamber.

### 2.7. Statistical Analysis

The data obtained for the analysis of all sperm variables were first assessed for normality and homoscedasticity by using Shapiro–Wilk and Levene tests respectively. A normal probability plot was used to assess normal distribution. In trying to obtain a normal distribution, data were transformed using acrsine square root (acrsin√x) before repeated-measures ANOVA was run. An ANOVA was applied to evaluate statistical differences in the distributions of observation (individual spermatozoa) within disposable and reusable counting chambers and then a generalized linear model (GLM) procedure was used to determine the effects on the mean kinematic values defining the different fields of disposable counting chambers ISAS^®^D4C depth. Differences between means were analyzed by a Bonferroni test. The statistical model used was:

Xijk = μ + Ai + Bj + AB (ij) + εijk(2)

where Xijk = the measured sperm motility variable, µ = the overall mean of variable x, Ai = the effect of depth, Bj = the effect of the counting chamber; AB (ij) = the effect of the interaction depth-counting chamber; and εijk = the residual.

Results are presented as mean ± standard error of the mean (SEM) Statistical significance was considered at *p* < 0.05. All calculations were performed using the IBM SPSS V.23.0 package for Windows (IBM Inc., Chicago, IL, USA).

## 3. Results

### 3.1. Optimum Frame Rate in Different Chambers

**Figure 2:**
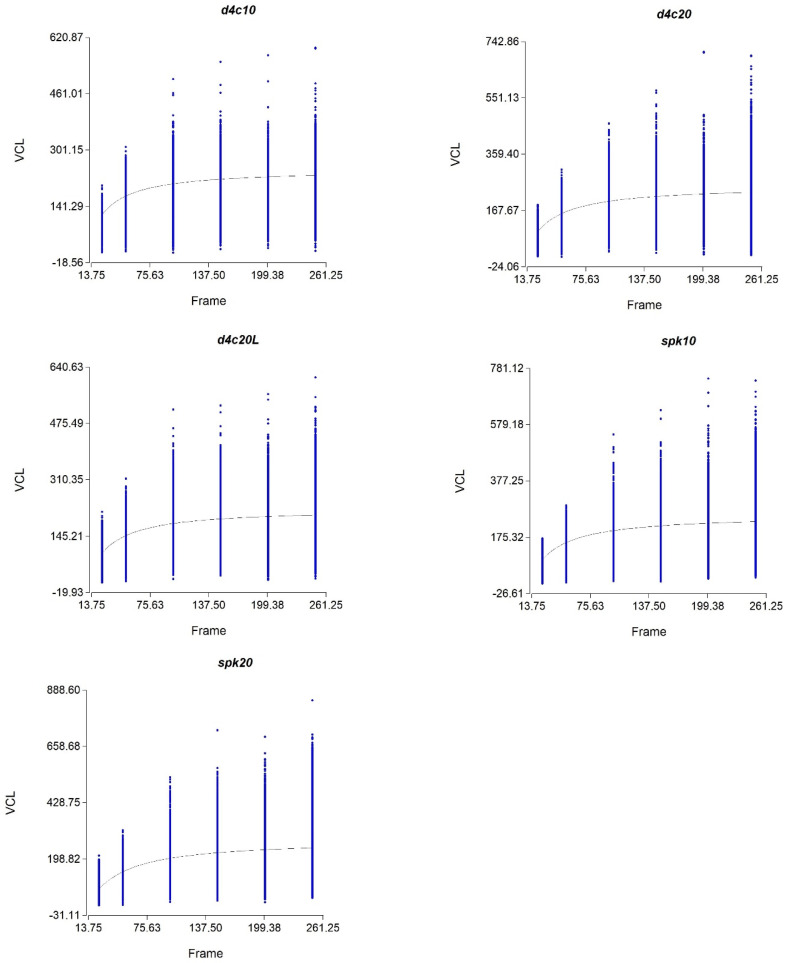
Sperm curvilinear velocity (VCL. μm/s) obtained at different frame rates in the different counting chambers.

The OFR was calculated for each chamber type (Figure 2). The higher OFR found was obtained by means of reusable chambers. However, OFR was higher in the chamber of 20 µm in depth compared to 10 µm in depth whatever the chamber type. Therefore, the highest value of the OFR was in Spk20 (278.4 fps) while the lowest was in D4C20L (225.3 fps) as presented in Table 1.

The OFR of all the chambers was between 225 f/s and 278 f/s. Thus, all the subsequent experiments were performed at 250 f/s for every chamber assuring a reliable measurement for sperm kinematic parameters in donkey semen.

**Figure 3:**
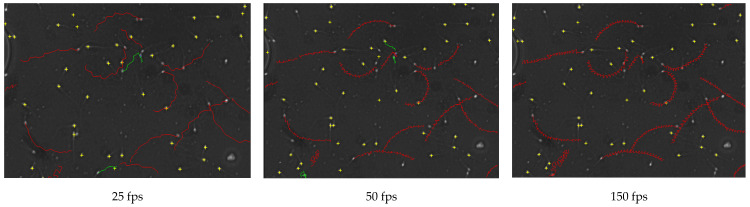
Donkey sperm motility tracks at different frames (25, 50 and 150), exhibiting 4 groups of spermatozoa velocity: rapid (red), medium (green), slow (blue) and static (yellow) in D4C10 chamber, fps = frames per second.

Sperm trajectory showed a different form as the FR increase. At a higher FR, the trajectory showed a much higher oscillation that was not possible to appreciate at lower FR as shown in Figure 3.

### 3.2. Effect of Chamber Type and Depth on Sperm Kinematic Parameters

The analysis of sperm kinematic parameters (VCL, VSL, VAP, LIN, STR, ALH) was generally characterized by a significantly higher value in reusable chambers compared to disposable chambers (*p* < 0.05). On the contrary, WOB and BCF were lower in reusable chamber Spk.

Then, when comparing the motility parameters in different depths for the same type and design chamber, a disposable D4C chamber of 10 µm depth reveals a higher value for the VAP, LIN, WOB and BCF rather than the D4C 20 µm depth chamber. Nevertheless, ISAS^®^D4C20L reusable chamber had the lowest value for all kinematic parameters except for WOB, STR and LIN which showed a higher value compared to D4C 20 µm (Table 2).

### 3.3. Effect of the Capture Field Inside the Counting Chamber

In D4C10, there was no clear tendency observed in the kinematic parameters as the spermatozoids travel from the point of deposition to the last field. However, the velocities (VCL, VSL, VAP) and BCF were higher in the last field while LIN increased along the counting way (form field 1 to 7) (Table 3).

The same results were found in the D4C20 chamber; we can observe an oscillatory change along the counting way. However, we observed a reduction in the velocity value (VCL, VSL, VAP) and the linearity in the last field (Table 3).

Regarding the D4C20L chamber, the highest values for all kinematic parameters (exception from the WOB) were observed closer to the point of deposition. However, the sperm had the lowest kinematic values in the middle of the chamber. The only value that showed no changes in the three counting zones was the BCF (Table 4).

Finally, in the reusable chambers, Spk spermatozoa showed similar behavior for both depths. In both Spk10 and 20 the sperm velocities (VCL, VSL, VAP) and ALH, values decreased significantly from the centre to the edges, while WOB and BCF increased (*p* < 0.05). Yet, VAP and LIN showed the highest value in the second counting ring in Skp20 (Table 5).

## 4. Discussion

In the present study, we attempted to standardize the technique used for the motility assessment of donkey sperm using high-resolution cameras and specific chambers.

The first step was to define the OFR for sperm kinematic analysis. Our work evidenced a direct positive relationship between capture frequency and VCL. Thus, capture frequency is the number of captures that permit the tracking of the sperm from one dot to another, hence we can reconstruct the sperm trajectory while VCL is the speed of the sperm (sperm trajectory/time) and dependent on sperm trajectory. As demonstrated in this study using non-linear regression, we could calculate the maximum frames needed, defined as OFR representing the threshold (α) where more captures will lead to the same sperm path. The OFR for the donkey sperm was different between chambers with a higher value in Spermtrack^®^ (278 fps) having a VCL of 248.36 µm/s. The lower frames were needed in the D4C20L chamber (225.7 fps). It has been noted in other studies in horses that the calculated OFR (309 fps) with a VCL is remarkably superior compared to the donkey (not published). Contrarily to what was found with lower frames (25 fps), the VCL was higher in donkeys compared to the horse [22]. This can be explained by the loss of information with a lower frame. It is believed that donkey sperm is faster than a stallion, but the current study showed less VCL with higher VSL and a straighter trajectory in donkey than the horse. Thus, this explains the lower OFR found in the horse compared to the donkey. Additionally, in recent studies, while using a high-performance camera and the same study design, depending on each species that the sperm shows non-linear trajectory will need more captures to define the correct track. This explains the differences obtained in the calculation of the OFR in the bull (256 fps) [24], the boar (212 fps) [25], and the salmon (250 fps) [26].

It was also found that the OFR is dependent on the chamber type and depth. In fact, the sperm motion or the flagellar beat is affected by the space where it is placed. In a similar study designed for bulls [24], this was also found in a different frame for different chambers.

The second step was to analyse the sperm behavior in different chamber types. As we can observe, the kinematic parameters were high in the reusable chamber compared to disposable chambers, except that the BCF and WOB values were higher in the disposable chamber. In fact, the same results were observed for VC, VSL and VAP on different species like horses, bulls, boars and bucks. Yet, the other parameters were variable depending on the species [24,27,28,29,30,31,32]. Those differences could be explained by the loading technique that could generate physical forces on the spermatozoa affecting its motility. As well, it was showed the existence of a certain interaction between sperm and ions of the glass mounted on the chamber, which would be toxic in certain species [14]. Furthermore, the chamber’s designs can exercise a certain force and affect sperm movement as seen in the D4C20L chamber where donkey sperm showed the lowest values for all kinematic parameters compared to other chambers which could be due to turbulence created inside this chamber [14]. The same results were found for the bull [33]. All these effects, as we can see, are specific to each species. So, it is essential to take these parameters into account for each species when choosing the chamber for sperm motility analysis.

The results of our study additionally demonstrated that the depth of the counting chamber influenced kinematic values. In fact, comparing the chambers of the same type for a different depth, we can observe that donkey sperm had greater VAP, VSL, LIN, STR, WOB and BCF in a narrow chamber of 10 µm rather than 20 µm chamber while VCL and ALH were greater in 20um chamber. The same result was found in other species such as the goat [27]. In the Belgian Blue bull and the Limousine bull breed [24], D4C10, 20 µm depths were used. This kind of linear motility movement is defined by low lateral amplitude and a high straight-line velocity. In actuality, it was found that this movement was essential for the sperm migration from the cervix to the uterus and then to the oviduct [34,35]. Moreover, this movement was also observed in the seminal plasma and the uterine fluids, with a low concentration of glucose. However, a study on the boar [36], the Holstein bull breed [24] and the stallion [29] determined that the sperm was faster (high VCL, VSL and VAP) with higher index values in the deepest chamber 20 µm. Yet, it has also been suggested that sperm kinematics are unaffected by the chamber depth [13]. Looking at the studies referred to above, it was observed that the frame rate setting and the chambers used to achieve different depths were not identical in all other respects. Therefore, differences in sperm kinematics could not be attributed solely to a chamber depth. A study conducted by [36], using lensless microscopy, showed that the kinetic parameters of boar semen in the deepest chambers of 100 µm increase significantly. Considering all these parameters discussed previously, it was demonstrated that the sperm behave differently depending on the chamber’s depth and the species. It is important to contemplate that there is a possibility that in the OFR with a higher chamber depth (more than the size of the sperm), the sperm will show different behavior.

To the best of our knowledge, this is the first work that studies the effect of the chamber field in the analysis of donkey sperm. The semen analysis demonstrated that the zone analysis in the chamber had a significant effect on the kinematic outcomes. The analysis in D4C10 rectangular chamber showed that the sperm increased in velocity as the drop moved to the last field. However, it was observed that the sperm lost its linearity. On the contrary, it was demonstrated that in the D4C20 chamber, the sperm showed opposite comportment for the velocity parameters. The same outcome was found in the fox samples [37] using the same chambers. It was suggested that the Segre–Silderberg effect altered the sperm movement as a consequence of the hydrodynamic drive of the fluid within the capillary-loaded chambers [38]. In this case, it affected the sperm tail [30,32] and the vitality [33]. In fact, we can observe that the results were emphasised in a narrow chamber when observing the sperm of the donkey. In the circle form chamber, Spermtrack^®^, the drop is moved by the force of the coverslip. In our study, it was found that when the drop moves from the centre to the edges, the motility decreased in both chambers’ depth, but the linearity increased. This result could be explained by the force of the fluid in the centre, which is generated by the cover that moves the sperm forward increasing the linearity but affecting the velocity. A similar result was found in the ram using a slide-coverslip [27,32]. However, [39] when using a Makler^®^ for the bull sperm, no differences between the centre and the edges could be determined [39]. As we can see, this could implicate the biology or metabolism of each species which behave differently [40].

Finally, it is important to also consider the dilution rate which had a direct effect on sperm motility parameters as observed in our study. The sperm velocity (VAP and VCL) and the progressiveness (BCF, STR and LIN) values were higher in the high-density sample (80 × 10^6^ sperm/mL) compared to a low density (30 × 10^6^ sperm/mL) sample for all the chambers. As previously reported in many studies, the semen concentration could affect sperm motility parameter values recorded by CASA [41]. The same results were found for different studies in dogs [42] and bulls [43] with a higher initial sperm concentration resulted in an increase in sperm velocity (VAP, VSL and VCL) and progressiveness. This difference can be related to the “dilution effect” referring to the detrimental effect on sperm quality, motility and resistance to a cold shock when adding a high volume of diluent of raw semen. The dilution effect was observed in a low sperm concentration (i.e., <20 × 10^6^ sperm/mL) and demonstrated in various species [44,45,46]. Nevertheless, a study on horses [45,47,48] and humans [49] using a lower sperm density (2.5 × 10^6^ sperm/mL) revealed higher motility parameters due to the minor effect that the “dilution effect” had on the sperm motility comparatively to other species. We propose a concentration of 80 × 10^6^ sperm/mL for further experiments in the study of the donkey when using a narrow chamber of 10 µm and especially in a disposable chamber. Usually, at high concentrations, the sperm aggregates affecting the CASA system results. This was reported for a concentration higher than 100 × 10^6^ sperm/mL [32]. Yet, in the present study, with a concentration of 80 × 10^6^ sperm/mL, the videos were clear, and the trajectories were defined correctly. On the other hand, in reusable chambers, we recommend using a lower concentration since the concentration of 80 × 10^6^ sperm/mL was too dense. Nonetheless, when using the playback facility for sperm tracking, the result showed a wrong trajectory reconstruction due to erroneous head detection in following frames, collision, and cross-tracks. Essentially, a different sperm concentration was proposed for CASA evaluation for different species [43,50,51] such as the horse where the concentration used is between (25 × 10^6^ and 50 × 10^6^ sperm/mL) [46,52]. The differences in the sperm concentration suggested for the analysis, in different studies, that it is likely due to the different usage of CASA devices and chambers and it is directly related to the species-specificities.

In the present study, a classical skim-milk extender (Kenney extender) was used. Rota et al. [53], by means of a previous CASA system, observed an effect of extender on sperm motility patterns of Amiata donkey spermatozoa. Further studies are needed to better understand the changes in motility patterns of donkey spermatozoa caused by semen extenders, with different composition and fluidity and using new CASA devices and chambers.

## 5. Conclusions

Each species, including horse and donkey, has its own sperm motility patterns; as a result, each species needs its own CASA system analysis conditions. These conditions are changing according to the constant improvement of related technologies. Current and new high-resolution video-cameras, informatic software and hardware-increased capacities (but limited) or new specific chambers to analyse sperm motility are changing previously defined sperm motility patterns. Then, to define the OFR, the kinds and depths of analysis chambers or the sample dilution are very important to more accurately describe the species-specific sperm motility. Thus, when examining the motility of donkey sperm, the 250 fps in Spk10 chamber and analyzing a minimum of nine fields considering all the capture area (centre and edges) and means determined at a concentration 30 × 10^6^ sperm/mL represents an excellent choice.

## Figures and Tables

**Table 1:** Optimum frame rate calculated to have the threshold level for different chambers types and depths. Curvilineal velocity of donkey sperm for the optimal frame rate (VCLα) and with different frames rates (25–250) calculated based on α and β values.

Chamber Type	Chamber	*n*	α	SEMα	β	SEMβ	VCLα	VCL25	VCL50	VCL100	VCL150	VCL200	VCL250
Disposable													
	D4C10	14,451	247.6	0.97	18.43	0.31	229.79	118.46	171.26	205.92	218.97	225.8	230.00
	D4C20	64,732	252.96	0.62	23.64	0.25	230.30	98.26	157.65	199.7	216.07	224.75	230.13
	D4C20L	76,812	225.71	0.49	21.18	0.18	207.72	96.74	147.76	182.62	195.98	203.02	207.37
Reusable													
	Spk10	105,679	255.01	0.55	24.23	0.19	231.89	96.74	157.07	200.13	216.97	225.91	231.45
	Spk20	172,918	278.46	0.46	31.80	0.15	248.36	78.04	147.41	202.60	225.26	237.52	245.20

*n*: number of spermatozoa analyzed; α = asymptote of curvilinear velocity; β = rate of increase; SEM = standard error of the mean; VCL = curvilinear velocity (μm/s). D4C10, D4C20: Disposable chambers 10 μm and 20 μm depth. D4C20L: Leja disposable chamber 20 μm depth. Spk10, Spk20: Reusable chambers (Spermtrack^®^) 10 μm and 20 μm depth.

**Table 2:** Motility parameters (mean ± SEM) of donkey spermatozoa determined by CASA system using disposable and reusable chambers obtained at 250 frames.

Chambers	Disposable			Reusable	
	D4C	D4C	D4CL	Spk	Spk
Depth	10 µm	20 µm	20 µm	10 µm	20 µm
VCL	227.91 ± 1.19 ^d^	238.16 ± 0.83 ^c^	223.77 ± 0.77 ^d^	260.47 ± 0.78 ^b^	268.07 ± 0.54 ^a^
VSL	70.19 ± 0.67 ^c,d^	71.16 ± 0.45 ^c^	68.93 ± 0.42 ^d^	83.57 ± 0.45 ^a^	80.46 ± 0.3 ^b^
VAP	181.57 ± 0.8 ^a^	177.18 ± 0.52 ^b^	168.15 ± 0.5 ^c^	183.8 ± 0.48 ^a^	181.66 ± 0.33 ^a^
LIN	29.82 ± 0.27 ^a,b^	28.3 ± 0.18 ^c^	29.32 ± 0.19 ^b^	30.23 ± 0.18 ^a^	28.46 ± 0.12 ^a,b^
STR	37.67 ± 0.38 ^d^	38.09 ± 0.25 ^d^	39.06 ± 0.24 ^c^	42.61 ± 0.23 ^a^	41.47 ± 0.16 ^b^
WOB	79.1 ± 0.19 ^a^	73.72 ± 0.13 ^c^	74.39 ± 0.15 ^b^	70.19 ± 0.13 ^d^	67.5 ± 0.09 ^e^
ALH	1.17 ± 0.01 ^e^	1.26 ± 0.01 ^c^	1.22 ± 0.01 ^d^	1.39 ± 0.01 ^b^	1.44 ± 0.0049 ^a^
BCF	42.58 ± 0.2 ^a^	40.35 ± 0.14 ^c^	38 ± 0.14 ^d^	40.79 ± 0.12 ^b^	39.96 ± 0.08 ^c^

SEM: Standard Error of the Mean; VCL (μm/s) curvilinear velocity; VSL (μm/s) straight line velocity; VAP (μm/s) average path velocity; LIN (%) linearity; STR (%) straightness; WOB (%) wobble; ALH (μm) amplitude of lateral head displacement; BCF (Hz) beat-cross frequency. Disposable chambers D4C 10 μm and 20 μm depth. Reusable chambers. Spk 10 μm and 20 μm depth. ^a,b,c,d^ Within columns, rates with different superscripts differed (*p* < 0.05).

**Table 3:** CASA motility parameters (mean ± SEM) in different fields of disposable chamber ISAS^®^D4C10 and 20 µm depth.

Chamber	VCL	VSL	VAP	LIN	STR	WOB	ALH	BCF
**D4C10**								
1	237.0 ± 3.3 ^a^	71.96 ± 1.69 ^b^	186.6 ± 2.71 ^a^	28.94 ± 0.56 ^b,c^	37.37 ± 0.71 ^b,c^	77.73 ± 0.39 ^b^	1.20 ± 0.01 ^a^	41.26 ± 0.67 ^a^
2	226.9 ± 3.1 ^a,b,c^	66.70 ± 1.59 ^b^	180.39 ± 2.56 ^a,b^	28.03 ± 0.53 ^c^	35.51 ± 0.67 ^c^	78.79 ± 0.37 ^a^	1.18 ± 0.01 ^a^	41.62 ± 0.63 ^a^
3	218.56 ± 4.22 ^c,d^	66.62 ± 2.16 ^b^	177.59 ± 3.47 ^a,b,c^	29.63 ± 0.72 ^b,c^	36.63 ± 0.91 ^b,c^	80.53 ± 0.5 ^a^	1.14 ± 0.01 ^a^	42.02 ± 0.86 ^a^
4	233.54 ± 3.11 ^a,b^	71.18 ± 1.6b	183.96 ± 2.56 ^a,b^	29.66 ± 0.53 ^b^	37.63 ± 0.67 ^b^	78.58 ± 0.37 ^a,b^	1.2 ± 0.01 ^a^	41.99 ± 0.63 ^a^
5	218.26 ± 4.22 ^c,d^	67.45 ± 2.16 ^b^	177.22 ± 3.47 ^a,b,c^	30.46 ± 0.72 ^a,b^	37.76 ± 0.91 ^b^	80.63 ± 0.5 ^a^	1.14 ± 0.01 ^a^	42.34 ± 0.86 ^a^
6	212.65 ± 4.09 ^d^	69.66 ± 2.1 ^b^	170.19 ± 3.37 ^c^	31.97 ± 0.7 ^a^	40.27 ± 0.88 ^a^	79.41 ± 0.49 ^a^	1.12 ± 0.01 ^a^	40.91 ± 0.83 ^a^
7	252.96 ± 16.8 ^a^	93.72 ± 8.61 ^a^	198.38 ± 13.83 ^a^	35.64 ± 2.87 ^a^	45.5 ± 3.61 ^a^	78.06 ± 1.99 ^a,b^	1.23 ± 0.06 ^a^	43.78 ± 3.42 ^a^
**D4C20**								
1	238.94 ± 2.64 ^b^	68.96 ± 1.32 ^c^	171.01 ± 2.04 ^b^	26.99 ± 0.4 ^c^	37.94 ± 0.51 ^b^	70.54 ± 0.27 ^c^	1.29 ± 0.01 ^b^	38.41 ± 0.48 ^c^
2	215.94 ± 2.58 ^c^	65.06 ± 1.29 ^d^	162.94 ± 1.99 ^c^	28.2 ± 0.39 ^b^	37.66 ± 0.5 ^b^	74.28 ± 0.26 ^a^	1.17 ± 0.01 ^d^	36.79 ± 0.46 ^d^
3	241.81 ± 2.51 ^b^	70.26 ± 1.26 ^b,c^	180.81±1.94 ^a^	27.7 ± 0.38 ^b,c^	37 ± 0.48 ^b^	74.31 ± 0.26 ^a^	1.27 ± 0.01 ^b^	40.52 ± 0.45 ^b^
4	243.05 ± 2.38 ^b^	72.97 ± 1.19 ^b^	182.02±1.84 ^a^	28.35 ± 0.36 ^b^	37.88 ± 0.46 ^b^	74.47 ± 0.24 ^a^	1.27 ± 0.01 ^b^	40.88 ± 0.43 ^b^
5	244.19 ± 2.71 ^b^	73.77 ± 1.36 ^b^	182.1±2.09 ^a^	28.7 ± 0.4 ^b^	38.39± 0.52 ^b^	73.99 ± 0.28 ^a^	1.28 ± 0.01 ^b^	39.7 ± 0.49 ^b,c^
6	253.55 ± 3.51 ^a^	78.47 ± 1.76 ^a^	184.84±2.72 ^a^	30.13 ± 0.53 ^a^	41.1 ± 0.68 ^a^	72.75 ± 0.36 ^b^	1.33 ± 0.01 ^a^	41.51 ± 0.63 ^a^
7	222.69 ± 3.91 ^c^	63.82 ± 1.96 ^d^	169.34 ± 3.02 ^b,c^	26.61 ± 0.59 ^c^	34.95 ± 0.75 ^c^	74.77 ± 0.4 ^a^	1.22 ± 0.01 ^c^	38.54 ± 0.71 ^c^

SEM: Standard error of the mean; VCL (μm/s) curvilinear velocity; VSL (μm/s) straight line velocity; VAP (μm/s) average path velocity; LIN (%) linearity; STR (%) straightness; WOB (%) wobble; ALH (μm) amplitude of lateral head displacement; BCF (Hz) beat-cross frequency. Disposable chambers D4C 10 μm and 20 μm depth. Reusable chambers. Different letters of the alphabet within columns indicate significant differences between different fields (*p* < 0.05).

**Table 4:** CASA motility parameters (mean ± SEM) in different fields of disposable chamber ISAS^®^D4C20L.

Capture Fields	VCL	VSL	VAP	LIN	STR	WOB	ALH	BCF
A	227.53 ± 1.89 ^a^	70.55 ± 0.95 ^a^	169.65 ± 1.49 ^a^	29.54 ± 0.3 ^a^	39.71 ± 0.38 ^a^	74.03 ± 0.19 ^b^	1.24 ± 0.01 ^a^	38.6 ± 0.37 ^a^
B	219.63 ± 1.65 ^b^	65.43 ± 0.83 ^b^	164.74 ± 1.3 ^b^	28.28 ± 0.26 ^b^	37.83 ± 0.33 ^b^	74.12 ± 0.17 ^b^	1.21 ± 0.01 ^b^	37.68 ± 0.32 ^a^
C	222.51 ± 1.5 ^b^	69.13 ± 0.76 ^a^	167.8 ± 1.19 ^a,b^	29.52 ± 0.24 ^a^	39.1 ± 0.3 ^a^	74.58 ± 0.16 ^a^	1.21 ± 0.01 ^b^	37.91 ± 0.29 ^a^

SEM: Standard error of the mean; VCL (μm/s) curvilinear velocity; VSL (μm/s) straight line velocity; VAP (μm/s) average path velocity; LIN (%) linearity; STR (%) straightness; WOB (%) wobble; ALH (μm) amplitude of lateral head displacement; BCF (Hz) beat-cross frequency. Different letters of the alphabet within columns indicate significant differences between different fields (*p* < 0.05). Capture fields for semen analysis proximal (A), medium (B), distal (C) as described in Figure 1.

**Table 5:** Mean (±SEM) of sperm kinetic parameters (VCL, VSL, VAP, LIN, STR, WOB, ALH and BCF) in donkey semen in reusable chamber Spermtrack^®^ (SpK) 10 and 20 µm depth.

Chamber and Capture Field	VCL	VSL	VAP	LIN	STR	WOB	ALH	BCF
**SpK10**								
A	274.11 ± 2.31 ^a^	88.37 ± 1.28 ^a^	188.94 ± 1.69 ^a^	30.33 ± 0.36 ^a,b^	43.8 ± 0.48 ^a^	68.53 ± 0.22 ^b^	1.45 ± 0.01 ^a^	41.35 ± 0.39 ^a^
B	257.82 ± 1.24 ^b^	81.41 ± 0.69 ^b^	182.23 ± 0.91 ^b^	29.6 ± 0.19 ^b^	41.61 ± 0.26 ^b^	70.3 ± 0.12 ^a^	1.38 ± 0.0045 ^b^	39.84 ± 0.21 ^b^
C	257.6 ± 1.63 ^b^	83.48 ± 0.91 ^b^	182.8 ± 1.2 ^b^	30.69 ± 0.25 ^a^	43.07 ± 0.34 ^a^	70.53 ± 0.15 ^a^	1.37 ± 0.01 ^b^	40.3 ± 0.28 ^b^
**SpK20**								
A	271.45 ± 1.76 ^a^	82.66 ± 0.93 ^a^	177.73 ± 1.25 ^b^	28.08 ± 0.26 ^b^	42.47 ± 0.34 ^a^	64.84 ± 0.15 ^c^	1.47 ± 0.01 ^a^	38.54 ± 0.29 ^b^
B	271.84 ± 1.11 ^a^	81.69 ± 0.59 ^a^	183.81 ± 0.79 ^a^	28.71 ± 0.16 ^a^	41.89 ± 0.22 ^a^	67.51 ± 0.1 ^b^	1.45 ± 0.0041 ^b^	39.42 ± 0.18 ^a^
C	263.56 ± 1.05 ^b^	77.72 ± 0.56 ^b^	180.56 ± 0.74 ^b^	27.94 ± 0.15 ^b^	40.27 ± 0.21 ^b^	68.18 ± 0.09 ^a^	1.42 ± 0.0038 ^c^	39.54 ± 0.17 ^a^

SEM: Standard error of the mean; VCL (μm/s) curvilinear velocity; VSL (μm/s) straight line velocity; VAP (μm/s) average path velocity; LIN (%) linearity; STR (%) straightness; WOB (%) wobble; ALH (μm) amplitude of lateral head displacement; BCF (Hz) beat-cross frequency. Different letters of the alphabet within columns indicate significant differences between different fields (*p* < 0.05). Capture fields for semen analysis central field (A), medium field (B), border field (C) as described in Figure 1.

## References

[1] Carneiro G.F., Lucena J.E.C., Barros L.D.O. (2018). The Current Situation and Trend of the Donkey Industry in South America. J. Equine Vet. Sci..

[2] Faccia M., D’Alessandro A.G., Summer A., Hailu Y. (2020). Milk products from minor dairy species: A review. Animals.

[3] Camillo F., Rota A., Biagini L., Tesi M., Fanelli D., Panzani D. (2018). The Current Situation and Trend of Donkey Industry in Europe. J. Equine Vet. Sci..

[4] Yılmaz O., Boztepe S., Ertuğrul M. (2012). The domesticated donkey: III-economic importance, uncommon usages, reproduction traits, genetics, nutrition and health care. Can. J. Appl. Sci..

[5] Varner D.D.D. (2008). Developments in stallion semen evaluation. Theriogenology.

[6] Giaretta E., Munerato M., Yeste M., Galeati G., Spinaci M., Tamanini C., Mari G., Bucci D. (2017). Implementing an open-access CASA software for the assessment of stallion sperm motility: Relationship with other sperm quality parameters. Anim. Reprod. Sci..

[7] Colenbrander B., Gadella B., Stout T. (2003). The Predictive Value of Semen Analysis in the Evaluation of Stallion Fertility. Reprod. Domest. Anim..

[8] McCue P.M. (2014). Breeding Soundness Evaluation of the Stallion. Equine Reproductive Procedures.

[9] Whitesell K., Stefanovski D., McDonnell S., Turner R. (2020). Evaluation of the effect of laboratory methods on semen analysis and breeding soundness examination (BSE) classification in stallions. Theriogenology.

[10] Broekhuijse M.L.W.J., Šostarić E., Feitsma H., Gadella B.M. (2011). Additional value of computer assisted semen analysis (CASA) compared to conventional motility assessments in pig artificial insemination. Theriogenology.

[11] Holt W.V., Cummins J.M., Soler C. (2018). Computer-assisted sperm analysis and reproductive science; a gift for understanding gamete biology from multidisciplinary perspectives. Reprod. Fertil. Dev..

[12] Yániz J.L., Silvestre M.A., Santolaria P., Soler C. (2018). CASA-Mot in mammals: An update. Reprod. Fertil. Dev..

[13] Canisso I.F., Panzani D., Miró J., Ellerbrock R.E. (2019). Key Aspects of Donkey and Mule Reproduction. Vet. Clin. N. Am. Equine Pract..

[14] Bompart D., García-Molina A., Valverde A., Caldeira C., Yániz J., De Murga M.N., Soler C. (2018). CASA-Mot technology: How results are affected by the frame rate and counting chamber. Reprod. Fertil. Dev..

[15] Contri A., Gloria A., Robbe D., De Amicis I., Carluccio A. (2012). Characteristics of donkey spermatozoa along the length of the epididymis. Theriogenology.

[16] Quartuccio M., Marino G., Zanghì A., Garufi G., Cristarella S. (2011). Testicular Volume and Daily Sperm Output in Ragusano Donkeys. J. Equine Vet. Sci..

[17] Carluccio A., Panzani S., Contri A., Bronzo V., Robbe D., Veronesi M.C. (2013). Influence of season on testicular morphometry and semen characteristics in Martina Franca jackasses. Theriogenology.

[18] Gacem S., Papas M., Catalan J., Miró J. (2020). Examination of jackass (*Equus asinus*) accessory sex glands by B-mode ultrasound and of testicular artery blood flow by colour pulsed-wave Doppler ultrasound: Correlations with semen production. Reprod. Domest. Anim..

[19] Moustafa M.N.K., Sayed R., Zayed A.E., Abdel-Hafeez H.H. (2015). Morphological and Morphometric Study of the Development of Seminiferous Epithelium of Donkey (*Equus asinus*) from Birth to Maturity. J. Cytol. Histol..

[20] Rota A., Puddu B., Sabatini C., Panzani D., Lainé A.L., Camillo F. (2018). Reproductive parameters of donkey jacks undergoing puberty. Anim. Reprod. Sci..

[21] Miró J., Flotats A., Rivera M., Ocaña M., Taberner E., Peña A., Rigau T. (2006). OC3 Morphometry Characterisation of Catalan Donkey Spermatozoa and Identification of Sperm Morphometric Subpopulations. Reprod. Domest. Anim..

[22] Miró J., Lobo V., Quintero-Moreno A., Medrano A., Peña A., Rigau T. (2005). Sperm motility patterns and metabolism in Catalonian donkey semen. Theriogenology.

[23] Kenney R.M. (1975). Minimal contamination techniques for breeding mares: Techniques and priliminary findings. Proc. Am. Assoc. Equine Pract..

[24] Bompart D., Vázquez R.F., Gómez R., Valverde A., Roldán E.R.S., García-Molina A., Soler C. (2019). Combined effects of type and depth of counting chamber, and rate of image frame capture, on bull sperm motility and kinematics. Anim. Reprod. Sci..

[25] Valverde A., Madrigal M., Caldeira C., Bompart D., de Murga J.N., Arnau S., Soler C. (2019). Effect of frame rate capture frequency on sperm kinematic parameters and subpopulation structure definition in boars, analysed with a CASA-Mot system. Reprod. Domest. Anim..

[26] Caldeira C., Hernández-Ibáñez S., Valverde A., Martin P., Herranz-Jusdado J.G., Gallego V., Asturiano J.F., Dzyuba B., Pšenička M., Soler C. (2019). Standardization of sperm motility analysis by using CASA-Mot for Atlantic salmon (*Salmo salar*), European eel (*Anguilla anguilla*) and Siberian sturgeon (*Acipenser baerii*). Aquaculture.

[27] Del Gallego R., Sadeghi S., Blasco E., Soler C., Yániz J.L.L., Silvestre M.A.A. (2017). Effect of chamber characteristics, loading and analysis time on motility and kinetic variables analysed with the CASA-mot system in goat sperm. Anim. Reprod. Sci..

[28] Basioura A., Tsousis G., Boscos C., Lymberopoulos A., Tsakmakidis I. (2019). Method agreement between three different chambers for comparative boar semen computer-assisted sperm analysis. Reprod. Domest. Anim..

[29] Hoogewijs M.K., De Vliegher S.P., Govaere J.L., De Schauwer C., De Kruif A., Van Soom A. (2012). Influence of counting chamber type on CASA outcomes of equine semen analysis. Equine Vet. J..

[30] Lenz R.W., Kjelland M.E., Vonderhaar K., Swannack T.M., Moreno J.F. (2011). A comparison of bovine seminal quality assessments using different viewing chambers with a computer-assisted semen analyzer. J. Anim. Sci..

[31] Valverde A., Arnau S., García-Molina A., Bompart D., Campos M., Roldán E., Soler C. (2019). Dog sperm swimming parameters analysed by computer-assisted semen analysis of motility reveal major breed differences. Reprod. Domest. Anim..

[32] Palacín I., Vicente-Fiel S., Santolaria P., Yániz J.L. (2013). Standardization of CASA sperm motility assessment in the ram. Small Rumin. Res..

[33] Gloria A., Carluccio A., Contri A., Wegher L., Valorz C., Robbe D. (2013). The effect of the chamber on kinetic results in cryopreserved bull spermatozoa. Andrology.

[34] Suarez S.S., Pacey A.A. (2006). Sperm transport in the female reproductive tract. Hum. Reprod. Update.

[35] Shalgi R., Smith T.T., Yanagimachi R. (1992). A Quantitative Comparison of the Passage of Capacitated and Uncapacitated Hamster Spermatozoa through the Uterotubal Junction. Biol. Reprod..

[36] Soler C., Picazo-Bueno J., Micó V., Valverde A., Bompart D., Blasco F.J., Álvarez J.G., García-Molina A. (2018). Effect of counting chamber depth on the accuracy of lensless microscopy for the assessment of boar sperm motility. Reprod. Fertil. Dev..

[37] Soler C., García A., Contell J., Segervall J., Sancho M. (2014). Kinematics and subpopulations’ structure definition of blue fox (*Alopex lagopus*) sperm motility using the ISAS® V1 CASA system. Reprod. Domest. Anim..

[38] Douglas-Hamilton D.H., Smith N.G., Kuster C.E., Vermeiden J.P.W., Althouse G.C. (2005). Capillary-loaded particle fluid dynamics: Effect on estimation of sperm concentration. J. Androl..

[39] Valverde A., Areán H., Fernández A., Bompart D., García-Molina A., López-Viana J., Soler C. (2018). Combined effect of type and capture area of counting chamber and diluent on Holstein bull sperm kinematics. Andrologia.

[40] Dresdner R.D., Katz D.F. (1981). Relationships of Mammalian Sperm Motility and Morphology to Hydrodynamic Aspects of Cell Function1. Biol. Reprod..

[41] Spiropoulos J. (2001). Computerized semen analysis (CASA): Effect of semen concentration and chamber depth on measurements. Arch. Androl..

[42] Rijsselaere T., Van Soom A., Maes D. (2003). Effect of technical settings on canine semen motility parameters measured by the Hamilton-Thorne analyzer. Theriogenology.

[43] Contri A., Valorz C., Faustini M., Wegher L., Carluccio A. (2010). Effect of semen preparation on casa motility results in cryopreserved bull spermatozoa. Theriogenology.

[44] Makler A. (1991). Sealed mini-chamber of variable depth for direct observation and extended evaluation of sperm motility under the influence of various gases. Hum. Reprod..

[45] Buss T., Aurich J., Aurich C. (2019). Evaluation of a portable device for assessment of motility in stallion semen. Reprod. Domest. Anim..

[46] Iguer-ouada M., Verstegen J.P. (2019). Evaluation of the “Hamilton thorn computer-based automated system” for dog semen analysis. Theriogenology.

[47] Hayden S.S., Blanchard T.L., Brinsko S.P., Varner D.D., Hinrichs K., Love C.C. (2015). Theriogenology The “dilution effect” in stallion sperm. Theriogenology.

[48] Varner D.D., Blanchard T.L., Love C.L., Garcia M.C., Kenney R.M. (1987). Effects of semen fractionation and dilution ratio on equine spermatozoal motility parameters. Theriogenology.

[49] Makler A., Deutch M., Vilensky A., Palti Y. (1981). Factors affecting sperm motility VIII. Velocity and survival of human spermatozoa as related to temperatures above zero. Int. J. Androl..

[50] Mortimer D., Goel N., Shu M.A. (1988). Evaluation of the CellSoft automated semen analysis system in a routine laboratory setting. Fertil. Steril..

[51] Neuwinger J., Knuth U.A., Nieschlag E. (1990). Evaluation of the Hamilton–Thorn 2030 motility analyser for routine semen analysis in an infertility clinic. Int. J. Androl..

[52] Verstegen J., Iguer-Ouada M., Onclin K. (2002). Computer assisted semen analyzers in andrology research and veterinary practice. Theriogenology.

[53] Rota A., Magelli C., Panzani D., Camillo F. (2008). Effect of extender, centrifugation and removal of seminal plasma on cooled-preserved Amiata donkey spermatozoa. Theriogenology.

